# On the interdependence of ambulatory and hospital care in the German health system

**DOI:** 10.1186/s13561-016-0132-4

**Published:** 2017-01-17

**Authors:** Tugba Büyükdurmus, Thomas Kopetsch, Hendrik Schmitz, Harald Tauchmann

**Affiliations:** 1 Universität Duisburg-Essen, Essen, Germany; 2 CINCH - National Research Center for Health Economics, Essen, Germany; 3 RWI - Leibniz-Institut für Wirtschaftsforschung, Essen, Germany; 4 Ruhr-Universität Bochum, Bochum, Germany; 5 Kassenärztliche Bundesvereinigung, Berlin, Germany; 6 Universität Paderborn, Paderborn, Germany; 7 Friedrich-Alexander-Universität Erlangen-Nürnberg, Findelgasse 7/9, Nürnberg, 90402 Germany

**Keywords:** Health care, Ambulatory care, Hospital care, Medical specialities, Instrumental variables

## Abstract

For some considerable time now the interface between ambulatory and hospital care has been mooted as a cause of inefficiencies in the German health system and there have been calls for a softening of the strict separation between the two sectors. This debate emphasizes the need for detailed empirical information on the interdependence between the two sectors. Using extensive administrative data at the level of the 412 German counties for the years 2007 to 2009 and a simultaneous equation model which allows the numbers of ambulatory and hospital cases to be mutually interdependent, we examine the connection between ambulatory and hospital specialist care separately for ten medical specialties. The results show that the interdependence of ambulatory and hospital services is far from homogeneous. The relationship depends, on the one hand, on the specialty and, on the other, on the direction of the effect observed. This heterogeneity needs to be taken into account for cross-sector needs-based planning.

## Background

Despite the aging society and the connected increase in demand for health services which in principle accompanies it, the German hospital market is still conspicuous for its overcapacities [1]. An international comparison shows that only Austria has a higher hospital bed density than Germany. While in Sweden 2.1 beds in acute hospitals for every 1000 inhabitants must suffice, in Germany there are 5.7 [2]. One reason for the overcapacities originates from progress in medical technology. Services which previously had to be performed in hospitals can now increasingly be carried out on an ambulatory basis. This is particularly true of Germany where, in contrast to most other countries, a recognised and accepted feature of the healthcare system means that specialist medical services can be performed both on an ambulatory basis by office-based doctors and on an inpatient basis by specialist physicians working in hospitals.

For some considerable time now problems associated with this dual provision of medical care have been the subject of extensive debate in Germany, with the controversy focussing on the interface between the ambulatory and the hospital sectors. The loudest calls are for care to be reorganised to avoid this strict separation which manifests itself in the German health service not only in two completely different remuneration systems for ambulatory and inpatient services but also in two different planning regimes. While hospital planning is the prerogative of the 16 German states, there exists a separate system of needs-based planning for ambulatory medical practices with rules set at the federal level. In their present form these two planning systems are mutually incompatible. To quote but one example: planning in the hospital sector is by beds while in the ambulatory sector the planning unit is the individual doctor. During the drafting of the Statutory Health Insurance Structure of Services Act (2012) the idea of overcoming this incompatibility with cross-sectoral needs-based planning was suggested. Although in the end no such system was introduced, the recently added paragraph *§*90a in the German *Sozialgesetzbuch*, allows for a joint committee to be formed to submit recommendations on cross-sector healthcare issues.

For the implementation and the functioning of a cross-sector needs-based planning it is essential to have information about the linkage between the provision of services in the two sectors, ambulatory and hospital. Only with this knowledge can a functioning cross-sector planning system be devised. The linkage between the provision of services in the ambulatory and hospital sectors of the health service is therefore the subject of this article.

To date there have been only few international studies which examine the connection between the ambulatory and hospital sectors. For most countries this is because their medical services are structured differently. For example, in some countries with what is known as the GP or Gatekeeper Model access to medical services is restricted by law. In this model the GP has the task of piloting the patient through the health service, authorising treatment by an office-based specialist or in hospital. This implies a purely complementary relationship between the two sectors. In the literature, this healthcare structure has been critically analysed both in respect of countries which are subject to these restrictions (cf. for example [3, 4]) and with a view to the introduction of this model in Germany (cf. for example [5, 6]).

In addition, there are some studies which analyse the relationships between GPs and the different specialists in both the public and the private sectors. An analysis of Italian data by Atella and Deb [7], for instance, finds a significant substitutive relationship between utilization in the two sectors. The relationships are estimated with the help of a simultaneous equation system. This is based on the assumption that GP consultations have an influence on the numbers of the specialists’ cases, but not the other way around. It is also assumed that the case numbers of the specialists in the public system have an effect on those of their colleagues in the private system but not vice versa.

A study by Adhikari [8] which concentrates on the relationship between ambulatory and hospital medical services to treat visceral leishmaniasis in Australia, provides evidence of a substitutive relationship between the two sectors. In the study, the elasticity of demand is first calculated separately for each sector. Subsequently, the relationship between the sectors is analyzed on basis of cross price elasticity. Another study by Fortney et al. [9] presents results from a natural experiment at the U.S. Department for Veterans Affairs, in which primary care services were increased in some districts but not in others. They look at the relationship between the utilization of GP services and those of ambulatory and hospital specialists. Taking into account the interdependence of the utilization of GP and all other medical services the study identifies a substitutive relationship between GP and office-based specialist care. However, the relationship between ambulatory and inpatient specialist services is neglected.

Kopetsch [10] examines the relationship between the ambulatory and hospital services provided in Germany in the year 2000, using data at the county level for the states of Bavaria, North Rhine-Westphalia and Saxony. Since the relationship varies by medical speciality, the analysis is carried out separately for ten groups of specialists. For this, he estimates an equation in which the ambulatory case numbers per inhabitant and further control variables explain the number of the hospital cases per inhabitant. Potential endogeneity problems in the study are not dealt with. The empirical analysis finds a complementary relationship for the specialities dermatology, ENT, paediatrics and orthopaedics while no significant relationship between ambulatory and hospital cases is detected for the other specialities.

Another strand of the literature discusses whether the parallel provision of services by the two sectors can be considered an important cause of inefficiencies and waste of resources (cf. for example [11–14]). On the one hand, a structure which duplicates specialists can lead to vertical competition between the office-based specialists and the hospitals. In the absence of incentives this competition can be counterproductive since the services are not delivered where they can be performed most cost-effectively. On the other hand, unnecessary costs may be incurred for repeated examinations if ambulatory specialist treatment leads to hospitalization. Looking at the service event in the ambulatory and hospital sectors, most German and international studies concentrate on potential inefficiencies at the interface between the two sectors. Himmel et al. [15], for example, examined the problem of the flow of information between hospitals and GPs and the consequent discontinuity of care. In order to test this, the authors concentrated on the medication administered on admission to hospital and provided considerable evidence of changes in the prescriptions during the stay in hospital. Hach et al. [16] show that hospitalization neither saves the ambulatory follow-up treatment nor is accompanied by a reduction in medication.

There are also some studies, which concentrate on the re-hospitalization rates as an important indicator of inefficiency between the two sectors. Therefore, the impact of transitional care interventions compared to standard hospital discharges are analyzed. As an example Weinberger et al. [17] studied the effect of such an intervention, which was designed to increase access to primary care after hospitalization. However, they found that the veterans in the intervention group had significantly higher rates of re-hospitalization and if readmitted also longer stays in hospital than veterans in the control group. On the other hand a study by Coleman et al. [18] found significant reductions in re-hospitalization rates and also lower mean hospital costs for for the patients in the intervention group. Despite of contrary findings regarding to care transition and rehospitalization, it confirms once more that it is worth to analyze the interface of the two sectors.

It is important to note that the studies cited above focus on very specific aspects of the interdependence of ambulatory and hospital care for which the nature of the interplay between the two sectors might be quite different. In other words, one may well find a complementary relationship for one specific situation and a substitutive relationship for the other.

The present analysis takes broader perspective, unlike the majority of the existing studies, we use administrative data that comprehensively covers the provision of inpatient and ambulatory care in Germany. The present paper expands the empirical evidence on the interdependence between the two sectors in Germany, focussing on how the provision of services in one sector relates to utilization in the other. It should be noted, however, that the present paper is only an exploratory study and cannot say anything about possible channels for positive or negative relationships between utilization in different sectors. Nor can the paper make a statement on efficiency in the system, as both individual level data and data on health outcomes are missing. Yet the results could serve as a starting point to dig deeper into potential reasons for the found relationships.

The results show a significant negative relationship for paediatrics and dermatology, with more specialist ambulatory cases leading to fewer hospital admissions. Considering the reversed direction, however, i.e. the impact of additional hospitalizations on the number of ambulatory cases, a significant positive connection can be observed for orthopaedics, gynaecology and otorhinolaryngology (ENT), with more hospital cases leading to additional ambulatory cases and a significant negative relationship for internal medicine. Furthermore we report a significant positive effect of GP cases on surgery and orthopaedic cases in hospital and also a significant positive relationship between ENT and GP cases for the reversed direction. Considering an increase in the number of all specialist cases the results show a significant decrease of GP cases and except for internal medicine and surgery, the same connection can be observed for the opposite direction.

The results of this study highlight the importance of a disaggregation of the data for the individual medical specialities, as they reveal a clear heterogeneity in the interdependences between the sectors. The relationship depends, on the one hand, on the medical speciality and, on the other, on the direction of the influence observed. Any possible cross-sector needs-based planning should take these linkages into account.

## Data and descriptive analysis

In the course of the analysis data from different sources are linked. The first of the two most important data sources is the accounting data of the KBV (National Association of Statutory Health Insurance Physicians), which contains in detail the ambulatory case numbers per medical speciality. This administrative data covers all infomation of utilization of medical services by members of the German statutory health insurance system, i.e. 90% of the population.^1^ The case numbers are aggregated at the level of the 413 counties (412 after two counties merged in 2009). Since this information is used to calculate the quarterly remuneration of the statutory health insurance doctors, these data can be assumed to be highly accurate as they are thoroughly checked for both administrative and computational errors. The cases per county refer to the patients’ place of residence rather than place of treatment. There are the separate case numbers for GPs and various specialities.

The second important source, the DRG statistics, provide details of the hospital cases, which are also based on the patients’ place of residence. This source is also a full survey of all cases in Germany. To provide an initial overview, we first examine purely descriptively the relationship between the utilization of ambulatory and hospital services in the years 2007, 2008 and 2009. A key aspect of the analysis is that the various medical specialties differ in their care structures and technical facilities, and thus also in the extent to which medical progress has made it possible to shift services into the ambulatory sector. The pattern of interdependences between the sectors may therefore be specific to each speciality and is, in consequence, analysed separately for each of ten specialist fields. Both data sources allow separate empirical analyses for ten specialities: ophthalmology, surgery, internal medicine, gynaecology, dermatology, ENT, paediatrics, neurology, orthopaedics and urology. Due to the special role which they can play in the utilization of specialist services, GPs are also included in the study. Table 1 gives an overview of the mean average values of the case numbers per county for the years 2007 to 2009 combined.

**Table 1:** Descriptive statistics: utilization of services

	Hospitalizations			Ambulatory cases		
	per 100.000 SHI insurees			per 1.000 SHI insurees		
	Mean	Min	Max	Mean	Min	Max
GPs				2,708	1,391	3,669
Ophthalmology	413	155	1,042	409	214	793
Internal Medicine	8,919	5,596	14,847	383	180	866
Paediatrics	1,475	1,076	2,679	373	121	684
Surgery	5,943	4,292	10,538	191	66	428
Urology	926	604	1,581	153	69	378
Orthopaedics	979	575	1,753	315	144	755
Gynaecology	2,930	2,177	5,449	635	315	1,606
ENT	822	466	1,234	237	101	424
Neurology	1,051	590	2,102	181	61	545
Dermatology	266	164	488	273	69	784
Observations	1.238			1.238		

Detailed descriptive statistics on the utilization of services can be found in the Appendix. They show that there is variation in the utilization of services not only between the regions but also from year to year. However, there is no systematic pattern of the relationship between ambulatory and hospital cases per speciality over time.

**Fig. 1:**
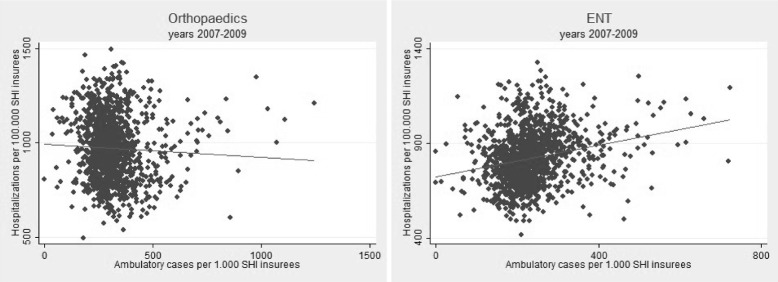
Scatter diagram of the descriptive relationship between ambulatory and hospital services per county. Note: The *gray line* represents the regression line

The heterogeneity between the counties can be clearly recognised. The average number of hospitalizations is up to seven times higher in some regions. In the ambulatory sector the average case numbers are in some regions up to eleven times higher than in regions with the lowest number of specialist cases. To illustrate the considerable regional variation Fig. 1 takes orthopaedics and ENT as examples of ambulatory and hospital cases. The gradient of the regression lines shows the respective direction of the correlation over all counties. Here the differences between the various medical specialities become apparent. While there is a positive correlation between hospital and ambulatory utilization in ENT the correlation in orthopaedics is negative. To provide a comprehensive summary of all medical specialities, the correlations of the case numbers in the two sectors are presented in Table 2.

**Table 2:** Correlations of the case numbers in the two sectors

	Correlation coefficient
	Total
Ophthalmology	0,2022^*^
Internal Medicine	0,0823^*^
Paediatrics	-0,1083^*^
Surgery	0,0567^*^
Urology	0,2553^*^
Orthopaedics	-0,1275^*^
Gynaecology	0,2415^*^
ENT	0,2257^*^
Neurology	0,0246
Dermatology	-0,0329

^*^Significant at the 5% level

Overall it becomes clear that the direction of the relationship crucially depends on the speciality. While for ophthalmology, internal medicine, surgery, urology, gynaecology, ENT and neurology there is a positive correlation, the correlation is negative in paediatrics, orthopaedics and dermatology. With the exception of neurology and dermatology, all correlations are significant.

However, the predominantly positive correlations still do not allow for drawing conclusions regarding a complementary relationship between ambulatory and hospital services. With a bivariate approach a positive correlation is to be expected everywhere since the average state of health of the respective population of the counties should be reflected in both measures. Counties with healthier inhabitants should in principle make fewer claims upon both ambulatory and hospital services. Equally, the demand for both types of medical service should be higher in counties with less healthy inhabitants.

### Further control variables

An important measure for determining the level of regional utilization is the need for medical services in a given region. It is therefore essential to control for variations in population structure and the state of health in each county. To capture the average state of health in a county we use both average life expectancy and the ’RSA risk factor’ devised by the Federal (Social) Insurance Office (*Bundesversicherungsamt* - BVA). The RSA risk factor is a measure of the average morbidity in a county. Used to calculate the compensatory transfers to those statutory health insurance funds whose membership evinces a higher risk structure, it summarizes as an index the morbidity of each county measured in terms of 80 important illnesses. This is a particularly attractive variable because it is objective, measuring the state of health as determined by doctors. At the same time, it is comprehensive and - like the dependent variables - probably highly accurate, as the information it provides determines the actual flow of money from the Risk Structure Compensation Scheme to the SHI funds.

In addition to health status, two other factors are relevant to our analysis. These are health behaviour and the efficiency of the individual’s own health production, as proposed by [19]. It is well-known, for instance, that levels of education and income correlate positively with healthy behaviour. To capture these factors, we use corresponding variables from the INKAR database maintained by the Federal Office for Building and Regional Planning (BBR) for 2007 and 2009.^2^ As there are no data available for 2008, we take the simplifying assumption that these structural variables remain constant over the short time span of two years. Specifically we take the average net monthly household income, the unemployment rate, the number of regional centres, the proportion of highly qualified and low-skilled workers, the shares of single-person households and immigrants, and the number of long-term care recipients per 10,000 inhabitants.

To measure the influence of environmental conditions, we take the annual average level of particulate matter pollution (PM10) ^3^ recorded at over 400 measuring stations distributed across Germany and published by the Federal Environment Agency (*Umweltbundesamt*). An algorithm has been used to translate the data to the county level. An overview of all control variables and their descriptive statistics can be found in Table 3.

**Table 3:** Descriptive statistics: control variables

	Mean	Std. Dev.	Min	Max
Health status				
Male life expectancy	77,077	1,460	72,200	81,100
Female life expectancy	82,103	1,020	77,900	84,900
RSA Risk Factor	1,009	0,071	0,847	1,204
Structural variables				
Proportion of population				
with SHI in %)	87,694	5,079	65,522	97,895
Long-term care recipients				
(per 10.000 population)	278,140	60,120	145,000	512,000
Unemployment rate in %	9,203	4,464	2,300	24,200
Net monthly household income	1.500,098	201,088	1.090,000	2.585,000
Number of large regional centres	0,401	0,564	0,000	4,000
Number of medium regional centres	2,219	2,149	0,000	11,000
Highly qualified workers in %	4,168	3,294	0,600	29,700
Low-skilled workers in %	14,400	4,825	6,400	39,500
Single-person households in %	36,290	4,379	20,400	55,800
Immigrants in %	7,152	4,536	0,700	25,200
Pollutants				
Particulate matter (PM 10)	19,737	6,112	0,000	31,000

## Methods

Any empirical analysis that is concerned with the interdependence of outpatient and inpatient care faces two methodological challenges. On the one hand, genuine interaction between the provision of services in the two sectors must be separated from correlation due to unobserved heterogenity, which might be an issue in the present data. In their analyses of the same data both Augurzky et al. [20] and Kopetsch and Schmitz [21] demonstrated that a large proportion of the regional variation in hospitalizations and physician consultations could be explained by observable differences in demographic structures and state of health. Nevertheless, a considerable part of the variation at county level remained unexplained and may have been caused by specific unobserved health or preference differences between the counties which could not be controlled for.

On the other hand, the interaction between two endogenous measures has to be considered. Utilization in the two sectors thus represents both an explanatory variable and a variable, which has to be explained. The analysis therefore requires the estimation of a simultaneous equation model whose parameters can only be identified with the help of instrument variables.

Finally, in any given region the supply of general practitioners (GPs) could play a role in the relationship between ambulatory and hospital cases, too. GPs typically refer their patients to office-based specialists, but will on occasion arrange for them to be admitted directly to hospitals. GP behaviour which differed regionally in this regard or regional variation in GP densities would thus impact on the linkage under examination.

In principle, there are two simple but contrasting hypotheses concerning the interplay of office- and hospital-based specialist care.

**First hypothesis (substitutes)**

It seems natural to regard ambulatory and hospital services as mutually substitutive. If medical services which can be rendered on either an ambulatory or an inpatient basis are performed by an office-based doctor, no hospitalization is required. The higher the physician density of the specialty or the higher the ambulatory cases, the lower the hospital cases in the region. Conversely, after a hospital stay the need for medical services should have been met without the need for corresponding ambulatory treatment. We hypothesize that this relationship exists for opthalmology, internal medicine, surgery, orthopaedics, neurology and dermatology. Our intuition is that this is due to the fact that several of the provided medical services in this diciplines can be performed on both ambulatory and hospital basis. An example for a medical service which is characteristic as a service that can be performed on ambulatory or hospital basis is cataract surgery.

**Second hypothesis (complements)**

It can also be argued that a complementary relationship exists. If, in a given region, ambulatory specialist treatment frequently leads to a hospital referral, and hospital stays necessitate ambulatory after-care, the result will be a positive correlation between office- and hospital-based specialist treatment. We expect a complementary relationship for paediatrics, urology, gynaecology and ENT. These medical specialities distinguish themselves from the other disciplines due to fact that several medical services can only be performed at a hospital while others can be carried out either at a hospital or on an ambulatory basis. Services which are characteristic for requiring a hospital stay are for example abdominal hysterectomy, thyroid surgery or an cochlear implant in case of deafness. This services are usually diagnosed by office-based doctors and require an admisssion to hospital, because of limitations in the ambulatory sector. There are different explanations for a complementary relationship.

1. A low density of specialists in the ambulatory care could increase the number of patients an individual doctor has to treat in a given period of time and consequently less diseases can be diagnosed due to less thorough examinations. Conversely, a high density of specialists allows the individual physician to spend more time with each patient. In this case more diseases could be diagnosed, which might lead to more hospital admissions.
2. Given a fixed treatment time per patient, a low physician density in a region would lead to less ambulatory cases or patients per office-based physician and thus to fewer hospital admissions than in an area with a higher physician density.
3. A high density of specialists in the ambulatory sector indicates high competition between the physicians. In this case the physician would rather refer the patient to a hospital than to another office-based doctor, as otherwise there would be the risk of the patient not returning to the referring physician. This argument also holds for GPs.

However, these attempted explanations show that the relationship may depend on the direction of the influence observed. This implies that in the present analysis the terms complements and substitutes are not used in accordance with their strict definitions used in microeconomic theory. There, two production factors can either be substitutes or complements but not both simultaneously. Deviating from this classical definition, here by complementary is meant that an increase in the number of cases in one sector results in an increased number of cases in the other, while substitutivity means that a reduced number of cases is the consequence. Rather than identifying the channels through which ambulatory and hospital cases influence each other, our contribution consists of illustrating the heterogeneity in the interdependence between the sectors over the various medical disciplines. This study is to be seen as a basis for further research, which needs to take the found linkages into account to organize a well-functioning cross-sector needs-based planning.

To explain the relationship between ambulatory and hospital case numbers, the following system of equations is formulated and estimated for each speciality separately:

1 $$\begin{array}{*{20}l} HC_{ij}=\alpha_{1}X_{i}+\beta_{1}HBD_{ij}+\gamma_{1}AC_{ij}+\delta_{1}GPC_{i}+\varepsilon_{1ij} \end{array} $$

2 $$\begin{array}{*{20}l} AC_{ij}=\alpha_{2}X_{i}+\beta_{2}PD_{ij}+\gamma_{2}HC_{ij}+\delta_{2}GPC_{i}+\varepsilon_{2ij} \end{array} $$

3 $$\begin{array}{*{20}l} GPC_{i}=\alpha_{3}X_{i}+\beta_{3}GPD_{i}+\gamma_{3}HC_{ij}+\delta_{3}AC_{ij}+\varepsilon_{3ij} \end{array} $$

where *HC*_*ij*_ denominates the number of hospital cases in speciality *j* per 100.000 inhabitants in county *i* ^4^, *AC*_*ij*_ the number of ambulatory cases in speciality *j* per 1.000 inhabitants in county *i*, *GPC*_*i*_ the number of GP cases per 1.000 inhabitants in county *i* and the *X*_*i*_ vector of control variables which contain, among other things, the average state of health in the county *i*. *HBD*_*ij*_ is the hospital bed density (beds per 100,000 inhabitants) in speciality *j*, *PD*_*ij*_ the physician density in speciality *j* and *GPD*_*i*_ is the GP density per 1,000 inhabitants in county *i*.

The equation system (1) to (3) thus allows ambulatory and hospital specialist utilization to be mutually interdependent. The GP care can be seen as both preceding and subsequent area. Thus the case numbers of GP care can also be influenced by specialist care. The particular aim of the analysis is the estimation of the coefficients *γ*_1_ and *γ*_2_, which model the nature of the interaction between hospital and ambulatory care, whereby positive values indicate a complementary and negative values a substitutive relationship.

We estimate the Eqs. (1) to (3) together with a three-stage least squares estimator (3SLS). For this the endogenous regressors *HC*_*ij*_, *AC*_*ij*_ and *GPC*_*i*_ in the equation system (1) to (3) are instrumented. Specifically they are explained by all exogenous variables in the equation system and the fitted values are used as regressors in the second stage. The correlations between the error terms of the three equations are then estimated from the residuals of this regression and used in the third stage of the estimation to increase the precision of the estimation results by a generalized least square estimation.

### Variables measuring the supply and exclusion restrictions

The utilization of services is closely linked to the supply in each of the sectors. A lack of availability in one sector could, in different regions, cause more services to be performed by specialists in the other. The analysis must therefore also consider the regional supply of hospital beds and office-based doctors.

In order to identify the model, we use exclusion restrictions. For instance the physician density (*PD*_*ij*_) directly affects the specialist cases (*AC*_*ij*_) but is excluded from Eq. (1). Thus, we assume that the physician density does not have an own effect on hospital cases once the supply of hospital beds and the specialist and GP cases are controlled for. Note that this does not rule out that physician density indirectly affects hospital cases via specialist case. The same holds for the GP density (*GPD*_*i*_).

Vice versa, hospital bed density (*HBD*_*ij*_) and GP density *GPD*_*i*_ are assumed not to have an own effect on specialist cases once specialist physician density as well as hospital and GP cases are controlled for. Thus, they are excluded from Eq. (2). Using similar arguments, we exclude *HBD*_*ij*_ and *PD*_*ij*_ from Eq. (3).

When utilizing medical services patients do not respect county borders. In order to model the regional supply of ambulatory treatment facilities actually available to patients more accurately, we therefore define the catchment area for each county as including not only that county itself but also all surrounding counties whose central point is no more than a 30-minute drive from the central point of the first county. By doing so, we obtain the physician density for a regional area which is of greater relevance for utilization patterns than the “county” as a purely administrative unit. The bed density (beds per 100,000 inhabitants) as a measure of the regional supply of hospital treatment facilities is determined for a geographical area which can be covered by car within 60 minutes. This takes account of the fact that patients are willing to cover longer distances to reach hospitals than to consult office-based doctors. The underlying data for this are provided by the Directory of Hospitals and Prevention or Rehabilitation Facilities [22].

## Results

Table 4 offers an overview of the average supply in the two sectors. Here, the previously mentioned, striking heterogeneity in the services available in the two sectors can be observed as well.

**Table 4:** Descriptive statistics: supply of ambulatory and hospital services

	Mean	Std. Dev.	Min	Max
Supply of hospital services per 100.000				
inhabitants within a radius of 60 minutes				
Bed density in ophthalmology	6,571	2,593	0,000	13,544
Bed density in internal medicine	192,611	30,886	97,041	306,750
Bed density in paediatrics	24,496	6,676	6,465	56,350
Bed density in surgery	133,667	19,133	72,922	253,616
Bed density in urology	18,935	4,079	1,640	32,310
Bed density in orthopaedics	30,399	11,081	0,000	105,177
Bed density in gynaecology	45,311	6,114	26,798	62,291
Bed density in ENT	14,799	4,596	0,000	37,291
Bed density in neurology	26,648	8,763	0,000	82,163
Bed density in dermatology	6,332	3,987	0,000	24,234
Supply of ambulatory services per 1.000				
inhabitants within a radius of 30 minutes				
Physician density for GPs	0,655	0,065	0,481	1,070
Physician density in ophthalmology	0,064	0,015	0,024	0,174
Physician density in internal medicine	0,108	0,039	0,015	0,323
Physician density in paediatrics	0,074	0,020	0,039	0,250
Physician density in surgery	0,056	0,020	0,015	0,199
Physician density in urology	0,033	0,008	0,013	0,076
Physician density in orthopaedics	0,064	0,018	0,018	0,149
Physician density in gynaecology	0,121	0,027	0,053	0,299
Physician density in ENT	0,047	0,013	0,015	0,134
Physician density in neurology	0,056	0,020	0,008	0,135
Physician density in dermatology	0,041	0,013	0,005	0,091
Observations	1.238			

Source: KHV, BAR (Federal Medical Register)

Table 5 show the results of the three-stage least square estimation for each speciality. Each of the ten columns refers to a regression with a different speciality. Besides the three regressors of prime interest to us, all control variables mentioned above were included in the regression and are to be found in the Appendix.

**Table 5:** Regression results for Eqs. (1), (2) and (3)

	Ophthalmology	Internal medicine	Paediatrics	Surgery	Urology
*Hospitalizations (1)*					
Specialist	0,251	-0,746	-0,751*	-0,918	1,660
Ambulatory Cases	(0,30)	(0,81)	(0,33)	(1,47)	(1,66)
GP Cases	−0,151	0,082	0,160	1,966***	0,200
	(0,10)	(0,53)	(0,13)	(0,52)	(0,29)
Bed Density	8,972***	14,882***	6,794***	12,009***	10,503***
	(1,78)	(1,08)	(1,09)	(1,26)	(1,61)
*Specialist Ambulatory Cases (2)*					
Specialist	−0,349	-0,041**	0,134	-0,024	0,043
Hospitalizations	(0,22)	(0,01)	(0,10)	(0,01)	(0,05)
GP Cases	−0,301**	−0,419***	−0,257***	−0,251***	−0,158***
	(0,09)	(0,11)	(0,05)	(0,06)	(0,03)
Specialist	1034,214**	1193,020***	1150,209***	1062,275***	353,974
Physician Density	(318,21)	(204,96)	(205,80)	(207,65)	(275,68)
*GP Cases (3)*					
Specialist	−0,576	−0,031	0,128	−0,047	0,199
Hospitalizations	(0,40)	(0,02)	(0,25)	(0,04)	(0,27)
Specialist	−1,139*	0,152	−1,427***	−0,045	−3,958*
Ambulatory cases	(0,55)	(0,24)	(0,34)	(0,54)	(1,61)
GP	587,778***	833,681***	621,521***	852,000***	340,511
Physician Density	(153,70)	(123,07)	(131,93)	(150,49)	(232,39)
	Orthopaedics	Gynaecology	ENT	Neurology	Dermatology
*Hospitalizations (1)*					
Specialist	−0,291	−0,107	0,834	−2,529	−0,189*
Ambulatory Cases	(0,27)	(0,49)	(0,49)	(1,66)	(0,09)
GP Cases	0,185*	0,271	−0,023	−0,062	−0,063
	(0,09)	(0,20)	(0,12)	(0,22)	(0,04)
Bed Density	4,538***	10,526***	6,823***	4,529***	1,877***
	(0,53)	(2,27)	(1,52)	(0,98)	(0,40)
*Specialist Ambulatory Cases (2)*					
Specialist	0,174*	0,320**	0,255***	0,083	−0,200
Hospitalizations	(0,07)	(0,12)	(0,06)	(0,07)	(0,56)
GP Cases	−0,200***	−0,353**	−0,137***	−0,124***	−0,306***
	(0,05)	(0,13)	(0,03)	(0,03)	(0,06)
Specialist	1097,432***	768,731*	557,960**	236,741*	1214,036***
Physician Density	(193,76)	(314,89)	(204,31)	(106,28)	(339,65)
*GP Cases (3)*					
Specialist	0,297	0,178	1,259**	0,569	−0,797
Hospitalizations	(0,21)	(0,23)	(0,45)	(0,33)	(1,31)
Specialist	−1,616***	−0,874*	−3,759***	−4,453***	−1,339**
Ambulatory Cases	(0,39)	(0,42)	(1,00)	(1,30)	(0,41)
GP	727,780***	646,518***	530,148**	451,625*	556,333***
Physician Density	(151,53)	(170,14)	(179,20)	(181,75)	(154,93)

Standard error in parentheses, * *p*<0,10,**p<0,05,***p<0,01

Note: All above mentioned variables were controlled for. Full results can be found in the Appendix

Where hospitalizations serve as a dependent variable (Eq. 1), we obtain for paediatrics and dermatology a substitutive while for the other specialities there is no significant connection. Examining the estimated effect of GP cases on the number of hospital cases reveals a significant complementary relationship for surgery and orthopaedics. While for no other specialty a significant connection is apparent, the sign of the estimated coefficients points in the same direction, with the exceptions of ophthalmology, ENT, neurology and dermatology. Yet for the latter three, the coefficient is almost zero. Disregarding statistical significance, for the majority of specialties the pattern points to extensive utilization of GP’s services increases the number of hospitalizations. One possible explanation is that more GP visits generate more opportunities for diagnosing diseases leading to hospital admissions. However, whether this interpretation is indeed correct could only be verified with the help of individual level data. The bed density and therefore the supply of hospital services has a positive influence on the hospital case numbers for all specialities. The service event in hospital care is therefore determined to a substantial degree on the supply side.

The results of the estimation of the equation with the ambulatory case numbers as a dependent variable (Eq. 2) indicate a significantly complementary relationship for orthopaedics, gynaecology and ENT. It is interesting that for some specialties more hospital cases lead to *more* ambulatory cases in these specialities whereas - looked at the other way around - more ambulatory cases lead to *fewer* hospital cases. There is therefore no clear interdependence between ambulatory and hospital cases here. This could be due to the fact that there are services in these specialities which are not feasible on an ambulatory basis and must therefore be performed in a hospital. Viewed the other way around, however, a hospital stay requires follow-up treatment in the ambulatory area and therefore manifests a complementary relationship. It should be noted, however, that most of the coefficients are not statistically significant.

Furthermore, all specialities are seen to have a substitutive relationship with the GP cases. Unlike specialist care in the hospital sector, more GP consultations do not lead to more cases for office-based specialists. Comprehensive GP care seems to obviate treatment by office-based specialists in many cases across all specialities. This result clearly argues against the hypothesis of initial GP visits often resulting in treatment cascades involving numerous subsequent specialist visits. For all specialities except for urology the physician density has a significantly positive influence on the case numbers. The supply side thus also plays an important role for the service event in ambulatory care.

The results of Eq. 3, where GP cases function as a dependent variable, we found a complementary relationship to ENT hospitalizations. This might be due to follow-up treatment that must not necessarily performed by a specialist, such as wound care. In accordance with this result, we find a qualitatively equivalent pattern of results concerning the link between ENT specialist ambulatory cases and GP cases (Eq. 2). Except for internal medicine and surgery, we also observe a substitutive connection for all specialties, which indicates a competitive relationship between GPs and office-based specialists.

With the help of F tests we examine whether the relevant instruments contribute significantly to the explanation of the instrumented variables. Table 6 shows that the instruments for all specialities have a significant explanatory power with regard to the variation in the specialist cases and all F tests are clearly over the critical rule-of-thumb limit of 10. This implies that the estimation results of the three-stage least square estimation are not the product of weak instruments and make an interpretation possible.

**Table 6:** F-Test of the instruments

	Ophthalmology	Internal medicine	Paediatrics	Surgery	Urology
Bed density for	F (1,1219) = 52,77	F (1,1219) = 168,14	F (1,1219) = 16,67	F (1,1219) = 130,49	F (1,1219) = 77,68
Specialists (radius 60)	Prob > F = 0,0000	Prob > F = 0,0000	Prob > F = 0,0000	Prob > F = 0,0000	Prob > F = 0,0000
Physician density for	F (1,1219) = 16,16	F (1,1219) = 35,30	F (1,1219) = 94,27	F (1,1219) = 50,94	F (1,1219) = 7,05
Specialists (radius 30)	Prob > F = 0,0001	Prob > F = 0,0000	Prob > F = 0,0000	Prob > F = 0,0000	Prob > F = 0,0080
Physician density for	F (1,1219) = 35,95	F (1,1219) = 47,74	F (1,1219) = 35,57	F (1,1219) = 51,39	F (1,1219) = 47,13
GPs (radius 30)	Prob > F = 0,0000	Prob > F = 0,0000	Prob > F = 0,0000	Prob > F = 0,0000	Prob > F = 0,0000
	Orthopaedics	Gynaecology	ENT	Neurology	Dermatology
Bed density for	F (1,1219) = 46,91	F (1,1219) = 42,29	F (1,1219) =78,34	F (1,1219) = 42,12	F (1,1219) = 13,44
Specialists (radius 60)	Prob > F = 0,0000	Prob > F = 0,0001	Prob > F = 0,0000	Prob > F = 0,0000	Prob > F = 0,0003
Physician density for	F (1,1219) = 78,28	F (1,1219) = 17,77	F (1,1219) = 64,05	F (1,1219) = 31,44	F (1,1219) = 51,03
Specialists (radius 30)	Prob > F = 0,0000	Prob > F = 0,0000	Prob > F = 0,0000	Prob > F = 0,0000	Prob > F = 0,0000
Physician density for	F (1,1219) = 31,51	F (1,1219) = 35,43	F (1,1219) = 47,25	F (1,1219) = 41,63	F (1,1219) = 42,36
GPs (radius 30)	Prob > F = 0,0000	Prob > F = 0,0000	Prob > F = 0,0000	Prob > F = 0,0000	Prob > F = 0,0000

All in all the results clearly suggest that, within the sector of ambulatory care, a substitutive relationship exists beween GPs and specialists visits. Our results are less clear with respect to the interaction of ambulatory and inpatient care. For several specialities no significant direct link in the number of cases is found in the data. Moreover, the few statistically significant effects are heterogeneous in their directions. All in all the results of this study highlight the importance of a disaggregation of the data for the individual medical specialities, as they reveal a clear heterogeneity in the interdependences between the sectors.

## Discussion

In Germany specialist care can be provided by both the ambulatory and the hospital sectors. The purpose of this study has been to examine the interdependencies between the two sectors. In doing so, we have taken account of the potential problem that the number of the specialist cases could be endogenous. That is to say, a complementary connection cannot be immediately inferred from a positive correlation, because in certain regions both ambulatory and hospital services could occur with particular frequency or infrequency due to unobserved factors, such as unobserved morbidity differences. Another problem with the potential to cause misleading interpretations of the correlation is that more hospital cases might also lead directly to more ambulatory cases.

The connections have been investigated by means of a simultaneous system of equations which not only controls for the regional differences in state of health and other structures but also takes into account the interdependent relationship between hospital and ambulatory cases. A strength of the study lies in its analysis of the treatments according to medical specialities, a procedure made possible by comprehensive administrative data sources. This differentiation by individual specialities clearly identifies relationships which would not be recognisable with less specific data, i.e. if ambulatory and hospital services were each treated as homogeneous goods. The heterogeneous basis of the analysis crystallises out the specific differences between the medical disciplines. It makes little sense to cast ophthalmological and gynaecological services together “into the same pot” since they are neither comparable nor mutually interchangeable. Finally, policy recommendations will also vary according to the speciality in question.

The results show a substitutive relationship for paediatrics and dermatology with more ambulatory cases leading to fewer hospital cases. If the perspective is reversed, however, and the focus is on how additional hospital cases influence case numbers in the ambulatory sector, a complementary relationship can be observed for orthopaedics, gynaecology and otorhinolaryngology (ENT). It is interesting that for some specialties more hospital cases lead to *more*/*fewer* ambulatory cases in these specialities whereas - looked at the other way around - more ambulatory cases lead to *fewer*/*more* hospital cases. Thus in this speciality there can be talk of neither a clearly substitutive nor an unambiguously complementary relationship. However, this interpretation mainly grounds in the point estimates as many of the estimated coefficients are not statistically significant.

The role played by general practice in all this is interesting. While GP consultations displace the services of ambulatory specialists across all disciplines, for hospital care the picture is different. Even it is just significant for surgery and orthopaedics the results indicate that additional cases for GPs seem to entail more hospital admissions for some specialities. Thus increasing the supply of GPs may well eliminate potentially superfluous specialist consultations, albeit at the price of raising the number of particularly cost-intensive hospitalizations. This might be due our theoretical argument of a substitutive relationship. The higher the competition between the physicians or the risk that a patient does not return to the referring physician the higher is the incentive to refer directly to the hospital.

On the other hand additional ambulatory cases in the specialities go along with smaller numbers of GP cases except for internal medicine.

The question as to whether ambulatory and hospital services complement or substitute each other depends, on the one hand, on the medical speciality and, on the other, on the direction of influence. Depending on the treatment needed, some patients can already be cared for entirely on an ambulatory basis or at least receive sufficient treatment from office-based doctors to avoid hospitalization. On the other hand, a hospital stay can necessitate intensive after-care treatment which is then provided by office-based doctors. Accordingly, the interdependence of ambulatory and hospital cases can vary in its intensity and even direction.

Our paper has several limitations which are grounded in the availability of data and should be taken into account when implications are considered. Given that our data are aggregated on the county level, we cannot clearly say, in case of a complementary relationship, whether patients first see a GP or ambulatory specialist and then enter a hospital or whether it is the other way around. Moreover, a complementary relationship per se is not informative about efficient or inefficient use of resources in the system. Positive relationships might either indicate that both sectors work well together, or hint at unnecessary duplications of services – and vice versa for substitutive relationships. Outcome data might be a way to evaluate this which, however, are not available in the present study.

Nevertheless, this exploratory study has its value in being a first starting point in a not well understood research area, and should be seen as a basis for digging deeper into the question of which deficits in the interplay of both sectors exist and how to improve the efficiency of health care provision in Germany. Heterogeneity in the interdependencies can also be identified on the aggregate level and the main policy implication is that this should be taken into account in cross-sector needs-based planning. This means, for example, that any reduction in capacity deemed necessary in the hospital sector should not be carried out indiscriminately across all medical specialities. Such cuts are better suited to departments whose services can be absorbed by the ambulatory sector than to those where they cannot.

## Conclusions

Again, while this study cannot make a statement on the current state of efficiency in the interplay between both sectors, it should be the starting point for further analysis, which focusses on implementing a well-functioning cross-sector needs-based planning regime. Especially for analysis of the committees, which were formed in individual states in 2012, to submit recommendations on cross-sector healthcare issues.

## Endnotes

^1^ Just over 10% of the German population are privately insured.

^2^ This database contains a total of some 500 indicators, based almost exclusively on official statistics, on topics such as population and social structure, the economy and employment, income and education, aggregated according to administrative areas (states, counties, local government districts). A precise description can be found at www.bbsr.bund.de.

^3^ *PM*_10_ (Particulate Matter 10) is a commonly-used standard specifying the aerodynamic diameter from which particles count as particulate matter. In the case of *PM*_10_ particles with a size of up to 15 microns go into a weighting function, in which at a size of approx. 10 micron half the particles go in.

^4^ Here and in the following “inhabitants” always means “statutorily insured inhabitants”. The term has been abbreviated for the sake of simplicity.

**Table 7:** Utilization of services (supplement to Table 1)

	Mean				Min	Max	Min	Max	Min	Max
	2007	2008	2009		2007	2007	2008	2008	2009	2009
Hospital cases per										
100.000 SHI population										
Ophthalmology	412	415	413		175	1118	164	1064	126	1023
Internal Medicine	8.720	8.952	9.084		5.564	14.233	2.967	14.973	5.409	15.335
Paediatrics	1.471	1.481	1.474		1.073	2.632	838	2.727	990	2.678
Surgery	5.804	5.962	6.062		4.049	10.054	3.088	10.703	3.932	10.856
Urology	910	931	938		601	1.532	371	1.535	464	1.677
Orthopaedics	947	985	1.006		495	1.713	536	1.798	600	1.763
Gynaecology	2.970	2.953	2.869		1.786	5.804	1.528	5.114	1.444	5.428
ENT	817	826	825		417	1.326	488	1.250	491	1.252
Neurology	1.016	1.045	1.092		558	2.055	397	2.169	614	2.082
Radiology	200	197	192		72	431	52	457	69	406
Dermatology	256	268	274		161	503	164	491	168	468
Ambulatory cases per										
1.000 SHI population										
General Practice	2.427	2.677	3.020		482	3.786	577	3.972	2.023	4.505
Ophthalmology	368	414	444		53	587	107	700	104	1.433
Internal Medicine	312	375	462		109	693	113	762	36	1.770
Paediatrics	303	461	355		28	480	65	722	59	961
Surgery	167	195	211		39	410	65	431	30	737
Urology	133	151	177		10	301	55	359	0	657
Orthopaedics	274	321	351		41	729	73	561	0	1.637
Gynaecology	564	647	693		149	859	152	967	100	3.551
ENT	214	240	257		14	417	73	461	0	722
Neurology	160	182	202		33	407	76	406	0	863
Radiology	148	233	288		9	509	93	544	0	1.879
Dermatology	245	278	295		65	546	81	457	0	1.544
N	413	413	412							

Source: DRG statistics, KBV

**Table 8:** Complete estimation results of the 3SLS: Eq. (1)

Specialist hospitalizations	Ophthalmology	Internal medicine	Paediatrics	Surgery	Urology
Specialist	0,251	−0,746	−0,751*	−0,918	1,660
Ambulatory Cases	(0,30)	(0,81)	(0,33)	(1,47)	(1,66)
GP Cases	−0,151	0,082	0,160	1,966***	0,200
	(0,30)	(0,81)	(0,33)	(1,47)	(1,66)
Bed Density	8,972***	14,882***	6,794***	12,009***	10,503***
	(1,78)	(1,08)	(1,09)	(1,26)	(1,61)
Male	−13,322	−251,611***	−49,505***	−55,089	16,414
Life Expectancy	(8,78)	(55,48)	(9,92)	(51,37)	(23,11)
Female	−13,904*	−252,349***	−7,400	−60,530	−26,399**
Life Expectancy	(6,87)	(47,11)	(10,23)	(37,80)	(8,51)
RSA Risk Factor	790,668***	9416,139***	59,333	235,517	16,951
	(239,02)	(1217,78)	(234,30)	(1032,70)	(719,47)
Long −Term Care Recipients	−0,342***	3,882***	0,146	1,446*	0,389**
per 10.000 Population	(0,10)	(0,73)	(0,14)	(0,58)	(0,13)
Unemployment Rate in %	−1,748	−57,284*	−4,133	−10,953	1,093
	(3,57)	(23,05)	(4,96)	(18,52)	(5,22)
Net Monthly	−0,026	−0,104	−0,133**	0,256	0,070
Household Income	(0,03)	(0,23)	(0,05)	(0,20)	(0,09)
Number of Large	26,504**	−42,441	10,944	−178,167***	−4,221
Regional Centres	(9,99)	(72,12)	(15,06)	(53,27)	(22,99)
Number of Medium	6,955***	56,546***	6,645**	21,394*	7,299***
Regional Centres	(1,55)	(11,04)	(2,08)	(8,94)	(2,00)
Highly Qualified	5,269*	2,472	13,874***	0,411	−0,910
Workers in %	(2,31)	(15,94)	(2,65)	(11,04)	(4,60)
Low −Skilled	−2,810	11,982	0,056	0,250	−1,716
Workers in %	(1,52)	(10,86)	(2,08)	(8,55)	(1,83)
Single −Person	1,040	−36,929***	−9,376***	−1,579	−5,070*
Households in %	(2,07)	(10,63)	(1,66)	(5,67)	(2,57)
Immigrants in %	−0,933	67,813***	10,472***	31,241***	5,143
	(2,45)	(11,09)	(1,83)	(8,60)	(5,34)
Particulate Matter (PM 10)	−0,575	1,047	−3,085*	−2,290	0,724
	(0,93)	(6,42)	(1,26)	(4,08)	(1,20)
Year 2007	−97,177	−341,588	41,458	971,194**	158,788
	(65,13)	(343,76)	(84,38)	(344,37)	(234,79)
Year 2008	−62,836*	−78,042	126,807***	642,815***	94,521
	(30,63)	(191,63)	(30,33)	(184,00)	(126,57)
Constants	2210,626**	36963,049***	5895,104***	6794,790	657,202
	(728,98)	(5216,84)	(840,37)	(4629,84)	(1990,99)
N	1238	1238	1238	1238	1238
Specialist	−0,291	−0,107	0,834	−2,529	−0,189*
Ambulatory Cases	(0,27)	(0,49)	(0,49)	(1,66)	(0,09)
GP Cases	0,185*	0,271	−0,023	−0,062	−0,063
	(0,09)	(0,20)	(0,12)	(0,22)	(0,04)
Bed Density	4,538***	10,526***	6,823***	4,529***	1,877***
	(0,53)	(2,27)	(1,52)	(0,98)	(0,40)
Male	−33,468***	28,139	−23,392*	−38,480*	−13,574***
Life Expectancy	(7,99)	(16,89)	(11,13)	(16,05)	(2,93)
Female	−17,195*	−1,670	4,022	−49,471***	0,148
Life Expectancy	(7,42)	(14,56)	(6,38)	(10,52)	(2,39)
Specialist hospitalizations	Orthopaedics	Gynaecology	ENT	Neurology	Dermatology
RSA Risk Factor	266,322	−1927,010***	28,083	1737,440*	420,579***
	(186,38)	(331,36)	(183,55)	(696,28)	(69,43)
Long −term Care Recipients	0,351**	−0,023	0,543***	−0,047	0,067
per 10.000 Population	(0,11)	(0,25)	(0,09)	(0,27)	(0,04)
Unemployment Rate in %	−9,086**	16,902*	−3,725	−13,861*	−3,579**
	(3,02)	(7,75)	(2,89)	(6,14)	(1,33)
Net Monthly	0,132***	−0,347***	−0,059*	−0,021	0,023*
Household Income	(0,03)	(0,08)	(0,03)	(0,07)	(0,01)
Number of Large	−4,828	−16,837	24,910*	35,340	3,563
Regional Centres	(9,79)	(21,50)	(12,05)	(26,27)	(4,09)
Number of Medium	10,657***	1,623	4,543**	5,120*	0,829
Regional Centres	(1,77)	(3,63)	(1,55)	(2,46)	(0,55)
Highly Qualified	0,213	30,002***	2,149	1,580	5,730***
Workers in %	(2,40)	(7,32)	(2,08)	(3,03)	(1,03)
Low −skilled	−4,231*	12,114***	2,948*	4,016	−3,712***
Workers in %	(1,67)	(3,35)	(1,50)	(3,68)	(0,56)
Single −Person	−0,587	4,102	−8,417***	6,293	−0,410
Households in %	(1,87)	(7,25)	(2,23)	(7,25)	(0,64)
Immigrants in %	5,108***	12,239***	0,695	6,908***	1,481*
	(1,54)	(3,50)	(1,61)	(2,08)	(0,67)
Particulate Matter (PM 10)	−0,664	−2,693	0,430	−4,217	−0,917*
	(0,83)	(2,21)	(0,68)	(2,59)	(0,37)
Year 2007	64,083	200,153	2,554	−170,225	−56,515*
	(61,50)	(142,10)	(84,09)	(184,27)	(25,84)
Year 2008	65,479*	143,838*	−4,045	−64,197	−22,976
	(30,37)	(65,05)	(41,37)	(86,16)	(12,86)
Constants	3928,720***	1463,968	2220,432*	6785,421***	1139,500***
	(716,27)	(1615,06)	(980,77)	(1693,01)	(316,57)
N	1238	1238	1238	1238	1238

Standard error in parentheses, * *p*<0,10,**p<0,05,***p<0,01

Note: All above mentioned variables were controlled for. Full results can be found in the Appendix

**Table 9:** Complete estimation results of the 3SLS: Eq. (2)

Specialists ambulatory cases	Ophthalmology	Internal medicine	Paediatrics	Surgery	Urology
Specialist Hospitalizations	−0,349	−0,041**	0,134	−0,024	0,043
	(0,22)	(0,01)	(0,10)	(0,01)	(0,05)
GP Cases	−0,301**	−0,419***	−0,257***	−0,251***	−0,158***
	(0,09)	(0,11)	(0,05)	(0,06)	(0,03)
Specialist	1034,214**	1193,020***	1150,209***	1062,275***	353,974
Physician Density	(318,21)	(204,96)	(205,80)	(207,65)	(275,68)
Male	−27,529**	−56,462***	−6,735	−29,130***	−12,677***
Life Expectancy	(8,62)	(12,14)	(7,12)	(5,48)	(3,33)
Female	1,893	−11,697	13,939**	3,563	3,268
Life Expectancy	(7,42)	(9,98)	(5,31)	(4,89)	(3,42)
RSA Risk Factor	963,151**	1374,248***	342,856**	499,031***	384,871***
	(293,05)	(292,08)	(105,36)	(108,87)	(85,45)
Long −Term Care Recipients	−0,185	0,102	−0,014	0,134	−0,054
per 10.000 Population	(0,14)	(0,15)	(0,08)	(0,07)	(0,05)
Unemployment Rate in %	−10,135**	−23,250***	−9,766***	−9,558***	−2,354
	(3,11)	(4,25)	(1,97)	(2,13)	(1,29)
Net Monthly	−0,057	−0,162***	−0,071**	−0,073**	−0,048***
Household Income	(0,03)	(0,04)	(0,02)	(0,02)	(0,01)
Number of Large	30,030*	53,129***	25,673***	14,811*	11,818**
Regional Centres	(12,01)	(12,92)	(6,70)	(6,72)	(4,31)
Number of Medium	1,734	3,204	0,044	1,700	0,177
Regional Centres	(2,15)	(2,50)	(1,35)	(1,19)	(0,90)
Highly Qualified	6,752**	10,888***	−0,687	2,167	2,318*
Workers in %	(2,47)	(2,76)	(2,11)	(1,37)	(0,96)
Low −Skilled	0,025	−1,163	2,103	−0,495	0,455
Workers in %	(1,77)	(2,22)	(1,23)	(1,11)	(0,74)
Single −Person	5,869***	4,935**	3,231*	−0,274	1,283*
Households in %	(1,23)	(1,72)	(1,46)	(0,75)	(0,57)
Immigrants in %	−7,687***	−4,667*	0,080	−2,726*	−3,074***
	(1,61)	(2,24)	(1,37)	(1,17)	(0,68)
Particulate Matter (PM 10)	−2,660***	−5,063***	−2,331***	−1,068*	−0,554
	(0,77)	(1,00)	(0,56)	(0,50)	(0,33)
Year 2007	−218,644***	−307,591***	−162,635***	−158,596***	−131,132***
	(58,23)	(65,29)	(31,99)	(32,08)	(20,08)
Year 2008	−90,597**	−128,691***	54,803**	−62,213***	−70,303***
	(33,17)	(36,82)	(19,07)	(18,68)	(11,68)
Constants	2473,987**	6143,137***	−61,622	2672,262***	988,383**
	(932,71)	(1190,85)	(750,33)	(526,49)	(349,18)
N	1.238	1.238	1.238	1.238	1.238
Specialist Hospitalizations	0,174*	0,320**	0,255***	0,083	−0,200
	(0,07)	(0,12)	(0,06)	(0,07)	(0,56)
GP Cases	−0,200***	−0,353**	−0,137***	−0,124***	−0,306***
	(0,05)	(0,13)	(0,03)	(0,03)	(0,06)
Specialists	Orthopaedics	Gynaecology	ENT	Neurology	Dermatology
ambulatory cases					
Specialist	1097,432***	768,731*	557,960**	236,741*	1214,036***
Physician Density	(193,76)	(314,89)	(204,31)	(106,28)	(339,65)
Male	−8,219	−27,768*	−8,234*	−5,971	−20,240*
Life Expectancy	(5,49)	(12,56)	(4,02)	(3,29)	(9,27)
Female	7,037	3,935	0,829	1,152	−3,750
Life Expectancy	(5,41)	(12,45)	(3,48)	(4,29)	(6,49)
RSA Risk Factor	314,001**	812,547**	176,453*	309,764***	515,128*
	(104,84)	(296,34)	(70,24)	(84,15)	(248,76)
Long −Term Care Recipients	−0,130	0,231	−0,150*	−0,165**	−0,033
per 10.000 Population	(0,08)	(0,19)	(0,06)	(0,05)	(0,10)
Unemployment Rate in %	−1,822	−16,971***	−1,541	−1,981	−9,565***
	(2,01)	(5,07)	(1,29)	(1,21)	(2,87)
Net Monthly	−0,037	0,016	0,004	−0,039**	−0,032
Household Income	(0,02)	(0,07)	(0,02)	(0,01)	(0,03)
Number of Large	9,840	29,436	8,303	13,189**	28,567***
Regional Centres	(6,82)	(16,41)	(4,79)	(4,05)	(8,38)
Number of Medium	−3,122*	2,186	−0,112	−0,717	0,237
Regional Centres	(1,43)	(2,97)	(0,88)	(0,87)	(1,55)
Highly Qualified	4,029**	2,648	1,101	0,760	8,692**
Workers in %	(1,49)	(5,40)	(1,02)	(0,92)	(3,21)
Low −Skilled	2,121	−3,070	0,381	1,883*	−1,545
Workers in %	(1,23)	(2,99)	(0,82)	(0,73)	(2,61)
Single −Person	4,448***	11,395***	4,792***	4,060***	4,092**
Households in %	(0,86)	(2,03)	(0,71)	(0,61)	(1,26)
Immigrants in %	−2,260	−8,041*	−1,560*	−0,813	−4,108*
	(1,18)	(3,25)	(0,72)	(0,78)	(1,79)
Particulate Matter (PM 10)	−1,374*	−2,727	−0,584	−1,413***	−2,948***
	(0,54)	(1,39)	(0,36)	(0,34)	(0,70)
Year 2007	−160,511***	−284,296***	−107,172***	−101,125***	−210,424***
	(30,80)	(75,82)	(19,82)	(17,88)	(40,46)
Year 2008	−65,327***	−113,216*	−46,860***	−45,597***	−91,659***
	(18,76)	(44,71)	(11,71)	(10,69)	(22,99)
Constants	374,894	1473,006	697,276	492,033	2609,837***
	(538,55)	(1294,81)	(376,95)	(402,82)	(745,61)
N	1.238	1.238	1.238	1.238	1.238

Standard error in parentheses, * *p*<0,10,**p<0,05,***p<0,01

Note: All above mentioned variables were controlled for. Full results can be found in the Appendix

**Table 10:** Complete estimation results of the 3SLS: Eq. (3)

GP cases	Ophthalmology	Internal medicine	Paediatrics	Surgery	Urology
Specialist Hospitalizations	−0,576	−0,031	0,128	−0,047	0,199
	(0,40)	(0,02)	(0,25)	(0,04)	(0,27)
Specialist	−1,139*	0,152	−1,427***	−0,045	−3,958*
Ambulatory Cases	(0,55)	(0,24)	(0,34)	(0,54)	(1,61)
GP	587,778***	833,681***	621,521***	852,000***	340,511
Physician Density	(153,70)	(123,07)	(131,93)	(150,49)	(232,39)
Male	−73,708***	−75,607***	−51,278*	−76,703***	−74,444***
Life Expectancy	(12,65)	(13,71)	(20,17)	(15,01)	(13,93)
Female	7,028	4,682	28,698*	8,096	17,241
Life Expectancy	(16,70)	(13,98)	(14,45)	(13,36)	(18,18)
RSA Risk Factor	2124,649***	1673,021***	1371,695***	1510,455***	1989,814***
	(383,09)	(349,39)	(227,19)	(259,30)	(400,82)
Long −Term Care Recipients	−0,163	0,317	0,054	0,290	−0,130
per 10.000 Population	(0,29)	(0,20)	(0,21)	(0,21)	(0,29)
Unemployment Rate in %	−26,618***	−24,958***	−27,944***	−28,589***	−18,148***
	(3,99)	(4,57)	(3,63)	(4,31)	(5,14)
Net Monthly	−0,158*	−0,138*	−0,213**	−0,158**	−0,247**
Household Income	(0,06)	(0,06)	(0,07)	(0,06)	(0,08)
Number of Large	76,274***	56,228***	76,514***	61,814***	68,596***
Regional Centres	(18,36)	(16,54)	(16,51)	(15,05)	(19,08)
Number of Medium	2,888	2,880	0,486	1,623	0,741
Regional Centres	(4,46)	(3,47)	(3,70)	(3,49)	(4,71)
Highly Qualified	12,158*	4,342	4,580	5,831	10,937*
Workers in %	(4,97)	(4,31)	(5,76)	(3,82)	(5,30)
Low −Skilled	−4,294	−6,206*	−1,475	−6,552*	−0,330
Workers in %	(3,76)	(2,88)	(3,31)	(3,04)	(4,39)
Single −Person	4,429	−9,315**	1,041	−6,593**	3,622
Households in %	(4,43)	(3,25)	(4,04)	(2,37)	(4,93)
Immigrants in %	−15,685***	−7,856**	−6,661*	−10,046***	−15,960***
	(3,58)	(2,68)	(3,30)	(2,44)	(3,63)
Particulate Matter (PM 10)	−2,988	0,739	−3,422	−0,179	−2,067
	(1,95)	(1,81)	(1,80)	(1,48)	(1,92)
Year 2007	−637,721***	−576,277***	−628,767***	−586,597***	−739,949***
	(31,80)	(32,89)	(27,37)	(29,41)	(68,96)
Year 2008	−316,683***	−312,976***	−148,151***	−312,622***	−399,917***
	(23,21)	(21,37)	(42,11)	(20,07)	(42,43)
Constants	6808,650***	7204,256***	3720,273	7229,778***	6118,658***
	(1247,22)	(1465,95)	(2109,81)	(1447,05)	(1458,26)
N	1.238	1.238	1.238	1.238	1.238
Specialist Hospitalizations	0,297	0,178	1,259**	0,569	−0,797
	(0,21)	(0,23)	(0,45)	(0,33)	(1,31)
Specialist	−1,616***	−0,874*	−3,759***	−4,453***	−1,339**
Ambulatory Cases	(0,39)	(0,42)	(1,00)	(1,30)	(0,41)
GP	727,780***	646,518***	530,148**	451,625*	556,333***
Physician Density	(151,53)	(170,14)	(179,20)	(181,75)	(154,93)
Male	−54,815***	−68,119***	−51,877**	−44,313*	−69,495***
Life Expectancy	(16,44)	(13,62)	(17,10)	(19,47)	(18,80)
Female	16,669	10,755	8,224	18,368	2,214
Life Expectancy	(15,86)	(16,83)	(17,78)	(23,83)	(15,48)
GP cases	Orthopaedics	Gynaecology	ENT	Neurology	Dermatology
RSA Risk Factor	1448,408***	1501,701***	1264,721***	1721,808**	1673,007**
	(265,12)	(368,27)	(264,22)	(661,84)	(513,41)
Long −Term Care Recipients	−0,100	0,321	−0,574	−0,713*	0,090
per 10.000 Population	(0,25)	(0,27)	(0,37)	(0,35)	(0,25)
Unemployment Rate in %	−19,411***	−30,104***	−15,279**	−15,843*	−27,920***
	(5,09)	(5,48)	(5,19)	(6,40)	(4,73)
Net Monthly	−0,150*	−0,122	−0,037	−0,235**	−0,119
Household Income	(0,07)	(0,11)	(0,08)	(0,08)	(0,07)
Number of Large	52,497**	66,471***	47,696*	79,434***	70,791***
Regional Centres	(17,75)	(19,28)	(21,61)	(23,87)	(17,62)
Number of Medium	−5,559	2,750	−1,995	−4,924	0,677
Regional Centres	(4,37)	(4,18)	(4,52)	(4,76)	(3,71)
Highly Qualified	10,878*	9,807	5,665	4,339	17,526*
Workers in %	(4,60)	(7,39)	(5,18)	(5,27)	(8,30)
Low −Skilled	−1,281	−6,163	−2,909	6,216	−7,735
Workers in %	(3,80)	(4,05)	(4,17)	(5,23)	(5,90)
Single −Person	4,837	6,460	18,482*	18,637**	2,506
Households in %	(3,59)	(6,70)	(7,23)	(6,47)	(3,88)
Immigrants in %	−9,329**	−12,246**	−11,197***	−8,582*	−10,296**
	(2,85)	(4,32)	(3,20)	(3,80)	(3,95)
Particulate Matter (PM 10)	−1,767	−2,564	−1,574	−5,803*	−4,060
	(1,68)	(2,11)	(1,91)	(2,71)	(2,09)
Year 2007	−661,415***	−656,445***	−686,662***	−702,031***	−635,668***
	(31,22)	(48,38)	(38,98)	(60,98)	(34,01)
Year 2008	−326,545***	−322,895***	−333,583***	−340,861***	−314,415***
	(22,87)	(27,16)	(26,65)	(32,32)	(22,89)
Constants	4492,968**	5784,788***	4400,430**	3203,080	7198,978***
	(1678,49)	(1554,32)	(1635,34)	(2502,86)	(1535,34)
N	1.238	1.238	1.238	1.238	1.238

Standard error in parentheses, * *p*<0,10,**p<0,05,***p<0,01

Note: All above mentioned variables were controlled for. Full results can be found in the Appendix

## Appendix
